# Elecsys^®^ AMH assay: Determination of Anti‐Müllerian hormone levels and evaluation of the relationship between superovulation response in Holstein dairy cows

**DOI:** 10.1002/vms3.1509

**Published:** 2024-06-18

**Authors:** Davut Koca, Ahmet Aktar, Ali Osman Turgut, Hakan Sagirkaya, Selim Alcay

**Affiliations:** ^1^ Faculty of Veterinary Medicine Department of Obstetrics and Gynecology Van Yuzuncu Yil University Van Turkey; ^2^ Faculty of Veterinary Medicine Department of Reproduction and Artificial Insemination Bursa Uludag University Bursa Turkey; ^3^ Faculty of Veterinary Medicine Department of Animal Science Siirt University Siirt Turkey

**Keywords:** AMH, cattle, embryo production, superovulation

## Abstract

**Background:**

Anti‐Müllerian Hormone (AMH) serves as a crucial parameter in assessing the reproductive herd life and ovarian reserve in cattle. Consequently, extensive research is conducted on AMH levels. Various measurement methods can be employed to determine AMH levels. However, to our knowledge, no study has been conducted on Holstein donors using the Elecsys^®^ AMH kit.

**Objective:**

This study was designed to determine AMH levels in donors utilising the Elecsys® AMH kit and to evaluate the relationship between superovulation response parameters and AMH levels.

**Methods:**

In this study, we measured the serum AMH levels of 36 cows using the Elecsys^®^ AMH automated assay before the superovulation protocol (1st sample) and FSH injections (2nd sample). The cows were categorised into three groups based on their AMH levels: low, medium, and high AMH.

**Results:**

Positive correlations were identified between AMH and parameters associated with superovulation response. The high AMH level group exhibited significantly greater numbers of corpus luteum, total embryos, transferable embryos, and grade 1 embryos compared to the medium and low AMH groups (*p* < 0.05) There was no significant difference between AMH levels before the superovulation protocol and FSH injections(*p* > 0.05). Body condition score and parity did not significantly affect AMH levels in cows (*p* > 0.05). Also, AMH cut‐off values for the number of corpus luteum, total embryo, and transferable embryos were detected as 234, 227, and 210 pg/mL, respectively.

**Conclusion:**

These findings demonstrate that a high serum AMH level has a positive influence on the superovulation response. AMH can be used as a reliable marker for the selection of donors in Holstein cows.

## Graphical abstract

1. AMH can be used as a reliable marker for the selection of donors in Holstein cows.

2. High serum AMH level has a positive influence on the superovulation response.

3. AMH cut‐off values for the number of corpus luteum, total embryo, and transferable embryos were detected as 234, 227, and 210 pg/mL, respectively.

4. AMH levels can be reliably measured during independent time points in adult cattle.

5. Body condition score and parity did not affect AMH levels in Holstein cows.

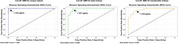

## 1 INTRODUCTION

Embryo transfer (ET) is an essential breeding technique employed to facilitate the cost‐effective production of high‐value calves and high‐performing cattle. This is achieved by utilising cows with exceptional genetic potential as donors. There is a noticeable uptick in the number of cows undergoing ET, signifying that this method has evolved into a prevalent and firmly established practice within the livestock industry (Mapletoft et al., 2002; Sagirkaya, 2009). Superovulation treatment, a pivotal step in the process of ET, entails the administration of hormones. This crucial procedure effectively retrieves multiple ova during a single estrous cycle. Nevertheless, there exists substantial variability between animals (Mapletoft et al., 2002). The variability in superovulatory response and the production of high‐quality embryos among donors poses a challenge in bovine embryo transfer programs. This variability arises from discrepancies in the size of follicular populations at the outset of superovulation treatment. Donors with larger follicular populations tend to yield a greater number of transferable embryos compared to those with smaller populations. Additionally, there exists a positive association between the number of ovarian antral follicles and various indirect indicators of fertility in cattle. These indicators encompass ovarian function (Ireland et al., 2007; Ireland et al., 2010; Jimenez‐Krassel et al., 2009), superovulation responses (Cushman et al., 1999; Kawamata 1994; Singh et al., 2004), in vitro blastocyst production (Pontes et al., 2009; Taneja et al., 2000) and herd longevity (Erickson et al., 1976). This underscores the significance of understanding and managing follicular populations to enhance the efficacy of bovine embryo transfer programs.

Anti‐Müllerian hormone (AMH) is a glycoprotein within the transforming growth factor beta family, produced by granulosa cells in healthy growing follicles (La Marca & Volpe, 2006). It plays a key role in overseeing the initial transition of primordial follicles into antral follicles. Additionally, it reduces the follicles' sensitivity to FSH during cyclic recruitment (Gigli et al., 2005; Yang et al., 2017). Anti‐Müllerian hormone (AMH) levels serve as a widely employed predictor for donor responsiveness to superovulation protocols in both cattle (Ireland et al., 2008; Ireland et al., 2010; Rico et al., 2009; Rico et al., 2011) and women (Hehenkamp et al., 2006; La Marca et al., 2010). AMH concentration remains relatively stable throughout reproductive cycles in ruminant species (Koca et al., 2024; Mossa & Ireland 2019; Turgut & Koca, 2024). It exhibits a positive correlation with the ovarian reserve (the total count of morphologically healthy follicles in the ovaries) in cattle (Ireland et al., 2010; Mossa & Ireland 2019), women (Peluso et al., 2014), goats (Karakas Alkan et al., 2020), sheep (McGrice et al., 2020) and mice (Kevenaar et al., 2006). Moreover, AMH is linked to the number of antral follicles that develop during ovarian follicular waves in cattle. Cattle with higher levels of circulating AMH tend to have elevated antral follicle counts (AFCs) and consequently larger ovarian reserves. They also possess a greater number of healthy, growing preantral and small antral follicles (Grimes et al., 1987). As a result, they exhibit more favourable responses to superovulation when compared to their age‐matched counterparts with lower AMH concentrations (Jimenez‐Krassel et al., 2009; 2015). These findings elucidate why numerous studies on both women (Broer et al., 2011; La Marca et al., 2010), and cattle (Morotti et al., 2018; Mossa et al., 2017; Mossa & Ireland 2019; Souza et al., 2015; Umer et al., 2019) also note a positive correlation between circulating AMH levels, and subsequent individual responses to superovulation. Moreover, there is strong evidence indicating that AMH exhibits a substantial degree of heritability (Nawaz et al., 2018; Walsh et al., 2014).

Anti‐Müllerian hormone (AMH) levels can be easily assessed from blood serum and plasma using various measurement methods and techniques. These measurements were conducted using various assays tailored for specific species: cats: canine AMH ELISA (AL‐116, Ansh Labs, Webster, TX, USA) (Claaßen et al., 2023); dogs: AMH Gen II ELISA (A79765, Beckman Coulter‐Immunotech s.r.o., Czechia) (Evci et al., 2023) and canine AMH ELISA (AL‐116, Ansh Labs, Webster, TX, USA) (Chotimanukul et al., 2023); mares: equine AMH ELISA (AL‐115, Ansh Labs, Webster, TX, USA) (Ucmak et al., 2023) and AMH Gen II ELISA (Beckman Coulter, Brea, CA, USA) (Fouché et al., 2022); cattle: AMH Bovine Test Kit, Enzyme‐Linked Fluorescent Assay (ELFA) method of the miniVIDAS^®^ (bioMérieux SA) (Sevgi et al., 2019); AMH Gen II ELISA (Beckman Coulter, Brea, CA, USA) (Furukawa et al., 2020); bovine AMH ELISA (AnshLabs®, Webster, TX, USA) (Widodo et al., 2022). In contrast to these kits, it has been documented that a fully automated Elecsys AMH assay (Roche, 56 for Cobas 601 platform) is utilised for dogs (Ozalp et al., 2023), camels (Seyedasgari et al., 2024) and heifers (Koca et al., 2023). Furthermore, the effectiveness of this kit has been confirmed in research carried out across various species, particularly in women. It serves as a diagnostic tool in accredited laboratories and studies about reproductive health (Anckaert et al., 2019; Deeks, 2015; Domain et al., 2022; Jacobs et al., 2019). However, as per our current knowledge, AMH levels have not been assessed in dairy cows using this particular assay, and no studies have been encountered that investigate the connection between AMH levels and superovulation in donor animals.

Considering the foregoing background, our objectives were as follows: (i) to determine AMH levels in donors utilising the Elecsys AMH kit, (ii) to assess the relationship between AMH levels at two distinct measurement intervals and (iii) to evaluate the relationship between superovulation response parameters and AMH levels.

## 2 MATERIAL AND METHODS

In this investigation, a total of 36 Holstein dairy cows from a single commercial dairy farm were designated as donors. These animals were selected as donors for the first time, and a superovulation protocol was administered to them for the initial occasion. The identical superovulation protocol was administered to the donor animals. The parity and body condition score (BCS) of cows ranged from 1 to 3 and 3 to 3.5, respectively. These cows underwent thrice‐daily milking and were housed in free‐stall barns. The ration followed the guidelines established by the National Research Council (NRC), and a comprehensive breakdown of the ration can be found in Table 1. Before the commencement of the study, thorough examinations were conducted on the ovaries and uterus. The reproductive organs of the donors were assessed through rectal palpation and real‐time ultrasonography (Hasvet 838, HASVET). Only animals with a healthy corpus luteum in the ovary and no abnormalities in the uterus and cervix were included in the study. Dairy cows exhibiting issues in their reproductive organs, such as adhesions, ovarian cysts, ovarian tumours, and metritis/pyometra, or showing the presence of fluid in the uterine lumen during ultrasonographic examination, were excluded from the study.

**TABLE 1:** Chemical composition of animal ration.

Nutrients	
Dry matter (DM)	24.5 kg
Crude protein of DM (%)	16.4
Ash of DM (%)	5.5
Starch of DM (%)	25.5
Non‐fibre carbohydrate of DM (%)	39
Neutral detergent fibre of DM (%)	32
Acid detergent fibre of DM (%)	19
Calcium of DM (%)	0.8
Phosphorus of DM (%)	0.4
Net energy lactation (Mcal/kg DM)	1.65

### 2.1 Collection and storage of blood samples for hormone analysis

Blood samples were collected before the superovulation protocol (before Prid insertion‐1st sample) and before FSH injections (2nd sample). Blood samples were obtained from the coccygeal veins of all animals. After centrifugation at 3000 rpm for 20 min, the sera were preserved in 1.5 mL Eppendorf tubes at −20°C till being assayed for AMH.

### 2.2 Assay of AMH

Serum samples were analysed for the AMH levels. Electrochemiluminescence immunoassay methodology was used to measure AMH levels. All samples underwent analysis under optimum conditions, utilising the Elecsys AMH automated assay (for Cobas 601 platform, Roche, Germany) as described earlier (Koca et al., 2023; Koca et al., 2024; Marchetti et al., 2023; Turgut & Koca, 2024). Before the primary trial, the samples underwent method validation as per the manufacturer's guidelines. Calibration and standard curves were analysed based on precise assigned values. The assay exhibited an analytical sensitivity of 0.01 ng/mL. The coefficients of variation for intra‐assay and inter‐assay ranged from 0.5% to 1.4% and 0.7% to 1.9%, respectively.

### 2.3 Superovulation treatment

At the outset, donor cows received intra‐vaginal progesterone (Prid Delta^®^, CEVA) alongside simultaneous intramuscular doses of GnRH (Receptal^®^, MSD), marking this as day 0. Starting from day 7, the cows underwent a series of intramuscular injections of FSH (100−100 µg, 75−75 µg, 50–50 µg, 25–25 µg; Stimufol) over four days, with 12‐h intervals. On the morning of day 9, an intramuscular dose of PGF_2α_ (Enzaprost^®^‐T, CEVA) was administered, and the Prid Delta was removed from the vagina the following evening. On day 11 artificial insemination was performed at least twice, following careful monitoring of the cows' estrus status. The number of corpora lutea (CL) on the ovaries was determined and recorded via ultrasound examination 7 days after artificial insemination. The identical superovulation protocol was administered to the donor animals.

### 2.4 Embryo collection

Embryos were retrieved from donors via uterine flushing, a non‐surgical technique, on the 7th day after artificial insemination (AI) (Alcay et al., 2022). This procedure began with the administration of epidural anaesthesia through a local anaesthetic injection (Adokain^®^, VERANO). Subsequently, a foley catheter was inserted through the cervix and secured by inflating the balloon within the uterus. A solution of 1 L of lactated Ringer's and 1.5% fetal calf serum (FCS) was employed as the flushing agent. Initially, the solution was introduced into two‐thirds of the uterine horn, after which it was subsequently aspirated through the filtration system. This process was repeated 4–5 times. The same flushing protocol was replicated on the opposite uterine horn. Following the uterine flushing, PGF_2α_ treatment (Enzaprost^®^‐T, CEVA) was administered intramuscularly to prevent pregnancy, and an intrauterine antibiotic (Fatroximin^®^, FATRO) was applied as a precaution against infections. Subsequently, embryos were identified utilising a stereo microscope, and then promptly transferred to a petri dish containing TL‐HEPES solution. Quality assessment of the embryos was conducted by the criteria outlined by the International Embryo Transfer Society (IETS, 1998).

### 2.5 Statistical analysis

Statistical analyses were performed using Minitab® (Version:  19.2020.2.0) and MedCalc (version: 22.016). For the sample size analysis, the power of the test was determined to be 80%, and the type‐1 error of 5% (G*Power statistics program, ver.3.1.9.4). The paired t‐ttest was utilised to compare AMH levels of animals before superovulation protocols (AMH‐1) and FSH injections (AMH‐2). Due to the lack of differences between AMH measurements, statistical analyses were performed using the first AMH measurement. One‐way analysis of variance (ANOVA) was performed to detect the effects of parity and BCS on the AMH level. Pearson correlation was utilised to detect correlations between AMH level and superovulation response parameters. Cows were categorised into three different groups using the AMH levels of animals of donors in the study. AMH levels were divided as follows; low (<132 pg/mL), medium (132–256 pg/mL), and high (>256 pg/mL). To detect AMH levels on superovulation response parameters, one‐way ANOVA test was performed. Receiver Operating Characteristic (ROC) analysis was performed to detect a serum AMH cut‐off value related to the number of corpus luteum, total embryo, and transferable embryo. Mean values for corpus luteum, total embryo, and transferable embryo were 14, 8, and 5, respectively. Therefore, these values were accepted as threshold to evaluate donors for each superovulation parameter. Interpretation of ROC analysis was based on the area under the ROC curve (AUC) and as follows: AUC ≤ 0.5 = poor, 0.5 < AUC ≤ 0.6 = accurate, 0.6 < AUC ≤ 0.7 = very Accurate, 0.7 < AUC ≤ 0.9 = highly accurate, 0.9 < AUC ≤ 1.00 = excellent. The statistical significance level was defined as *p* < 0.05.

## 3 RESULTS

Descriptive statistic values for AMH levels are given in Table 2. There was no significant difference between AMH levels before the superovulation protocol and FSH injections (*p* > 0.05).

**TABLE 2:** Descriptive statistic values of AMH (pg/mL) levels before superovulation and FSH injection.

	*n*	Mean ± SEM	Median	Minimum	Maximum	Q1–Q3
Before superovulation	36	223.3 ± 16.8	228.5	94.0	432.0	127.8–298.3
Before FSH injection	36	223.9 ± 16.7	226.0	96.0	425.0	129.3–302.5

Q1, first quartile; Q3, third quartile.

**FIGURE 1:**
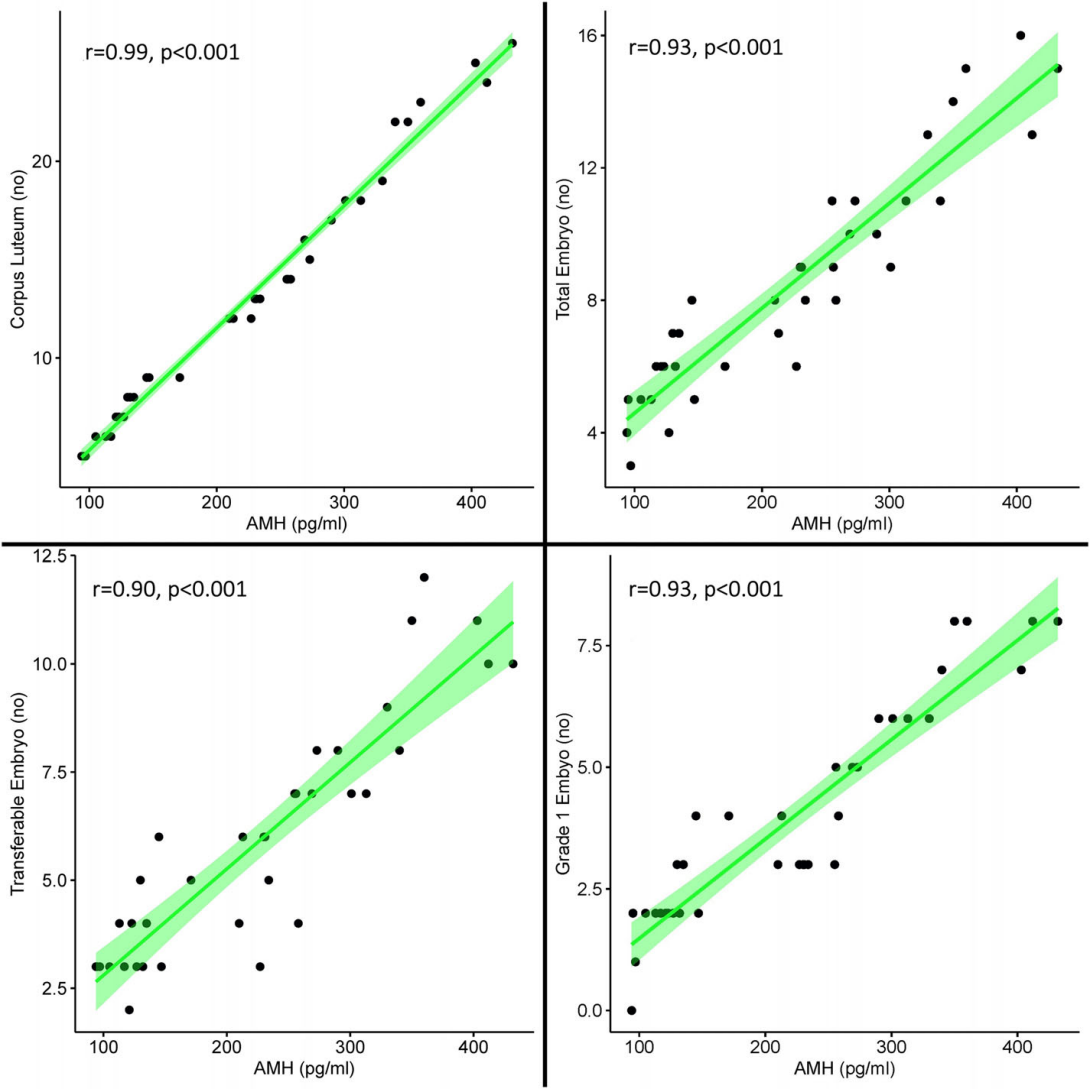
A scatter‐plot visualising correlations between AMH and superovulation response parameters.

Parity and BCS did not significantly affect the AMH level of cows (*p* > 0.05) (Table 3). Figure 1 shows scatter plots visualising correlations between AMH and superovulation response parameters.

**TABLE 3:** The effects of parity and body condition score on AMH levels.

Parity	*n*	Mean ± SD	*p* Value
1	13	235.1 ± 107.1	>0.05
2	12	212.4 ± 97.1	
3	11	224.3 ± 105.7	
BCS			
3	12	227.8 ± 108.9	>0.05
3.25	13	210.9 ± 102.8	
3.5	11	233.0 ± 96.7	

**FIGURE 2:**
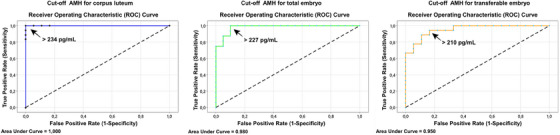
ROC curves and AMH cut‐off values for corpus luteum, total embryo, and transferable embryo. The arrow indicates the cut‐off point maximising the Youden index.

The number of corpus luteum, total embryo, transferable embryo, and grade 1 embryo was significantly greater in cows with high AMH levels than in cows with medium and low AMH levels (*p* < 0.05) (Table 4). Table 5 indicates the ROC analysis results. Figure 2 shows ROC curves for predicting superovulation parameters in Holstein cows. AMH cut‐off values for the number of corpus luteum, total embryo, and transferable embryos were detected as 234, 227, and 210 pg/mL, respectively (Figure 2).

**TABLE 4:** The effects of AMH level on superovulation response in dairy cows.

	AMH (pg/mL)		
	Low (115.75 ± 4.3)	Medium (214.8 ± 11.5)	High (339.4 ± 15.7)
*n*	12	12	12
Corpus luteum (no)	6.50 ± 1.16^c^	12.00 ± 1.90^b^	20.41 ± 3.70^a^
Total embryo (no)	5.33 ± 1.23^c^	7.83 ± 1.64^b^	12.33 ± 2.30^a^
Transferable embryo (no)	3.33 ± 0.77^c^	5.16 ± 1.40^b^	9.00 ± 1.75^a^
Degenerated embryo (no)	2.00 ± 1.12^b^	2.66 ± 1.15^ab^	3.25 ± 1.05^a^
Grade 1 embryo (no)	1.91 ± 0.79^c^	3.41 ± 0.79^b^	6.66 ± 1.15^a^

*Note*: Different superscript letters show statistically significant differences between mean values of low, medium, and high AMH levels. Values refer to mean ± SD.

**TABLE 5:** ROC analysis results of superovulation parameters for AMH.

	Sensitivity (%)	Specificity (%)	Youden index	Area under curve
Corpus luteum	100	100	100	1.00
Total embryo	100	90	90	0.98
Transferable embryo	94.44	83.33	77.77	0.95

## 4 DISCUSSION

Embryo transfer stands as a pivotal tool in reproductive practices, serving to enhance the genetic merit of quantitative traits in dairy cattle. However, the efficacy of this procedure depends on the superovulation performance exhibited by the donor cows. In cattle breeding, AMH is accepted as a crucial indicator of superovulation response. Studies indicate the correlation between superovulation response and AMH levels in Holstein dairy cows (Alward & Bohlen, 2020; Nabenishi et al., 2017; Souza et al., 2015; Umer et al., 2019). Elecsys AMH kit is used to determine AMH levels in heifers (Koca et al., 2024), sheep (Turgut & Koca, 2024), bitches (Ozalp et al., 2023), queens (Lapuente et al., 2023) and mares (Papas et al., 2021). This is the first report demonstrating a link between AMH levels and the superovulation response of donor cows, using the Elecsys AMH kit.

In Holstein calves, variations in AMH levels are observed during the pre‐pubertal phase following parturition. However, it has been documented that post‐puberty, AMH levels remain consistent (Mossa et al., 2017). Significantly, in cattle, AMH concentrations exhibit minimal fluctuations throughout the estrous cycle. Extensive research has firmly established a robust association between AMH measurements taken at any point within the cycle, both in heifers and cows, as well as those obtained on different days within the same or subsequent cycles (Ireland et al., 2009; Pfeiffer et al., 2014; Rico et al., 2009; Souza et al., 2015). These collective findings underscore the reliability of determining AMH concentrations using a single blood sample, irrespective of the specific day within the cycle, in cattle (Mossa & Ireland, 2019). In this study, no statistical difference was observed between the AMH levels during the two different sampling times regardless of the days of the cycle. This demonstrates that AMH levels can be reliably measured during independent time points in adult animals similar to earlier studies.

We conducted an examination of serum AMH levels in cows across various parity groups and BCS falling within the range of 3 to 3.5. The analysis revealed that neither parity nor BCS exerted a discernible influence on serum AMH levels in Holstein dairy cows. This aligns with prior studies conducted by Pfeiffer et al. (2014), Hirayama et al. (2017), and Koca et al. (2023), not only in Holstein but also in Simmental cattle. Thus, our findings substantiate and corroborate the conclusions of other studies conducted in cattle. Consequently, it was established that the parity and BCS of the donors included in our study bore no significant impact on AMH levels (Table 3).

There are many factors that affect superovulation in cattle. The main determinants include breed, age, superovulation protocol, hormones, nutrition, and ovarian reserve (Aziz et al., 2017; Besenfelder et al., 2020; Cizmeci et al., 2022; Estrada‐Cortés et al., 2019; Jelani et al., 2022; Mikkola et al., 2020; Reddy et al., 2023). In this study, donor animals were planned to be under the same breed, superovulation protocol, and nutritional conditions. This enabled to reliable assessment of the relationship between AMH levels and the superovulation response in Holstein donor cows.

The main parameters such as the number of corpus luteum, total embryos, transferable embryos, grade 1 embryos, and degenerated embryos play pivotal roles in assessing the response to superovulation in dairy cattle. Notably, there exist significant variations in both superovulation response and embryo quality among different donors, primarily arising from differences in ovarian reserves. Cows with higher ovarian reserves yield a greater number of transferable embryos after uterus flushing (Aziz et al., 2017). Anti‐Müllerian Hormone (AMH) emerges as a crucial indicator of ovarian reserve in cows. While the serum AMH level tends to remain stable post‐puberty, there are notable distinctions in AMH levels among individuals (Nabenishi et al., 2017). Research indicates that cows with higher serum AMH levels produce approximately twice as many transferable embryos compared to those with lower AMH levels (Aziz et al., 2017). Similar findings have been observed in cows and heifers across diverse breeds (Guerreiro et al., 2014; Ghanem et al., 2016; Hirayama et al., 2017). Additionally, studies have reported positive correlations in relation to serum AMH levels across various cattle breeds (Baruselli et al., 2018; Souza et al., 2015). Collectively, these findings underscore a significant association between serum AMH levels and the response to superovulation in cows (Table 4).

In this study, we observed significant (*p* < 0.001) and positive correlations between serum AMH levels and superovulation parameters (Figure 1). This aligns with findings from prior research. Hirayama et al. (2017) noted a significant correlation between serum AMH levels and the total number of embryos in Japanese Black cattle. Similarly, Sevgi et al. (2019) reported a positive and statistically significant correlation (r = 0.68) between serum AMH levels and both corpus luteum count and the number of transferable embryos. Souza et al. (2015) observed a positive correlation (*r* = 0.65) between corpus luteum count and serum AMH levels in high‐producing dairy cows. Likewise, Aziz et al. (2017) identified positive and significant correlations between serum AMH levels and parameters related to superovulation response in Holstein cows. Consequently, it can be deduced that a higher serum AMH level is associated with an enhanced superovulation response in dairy cows.

In ROC analysis, we have determined AMH cut‐off values for corpus luteum, total embryo, and transferable embryo as 234, 227, and 210 pg/mL, respectively. Hirayama et al. (2017) reported AMH cut‐off for >15 ova/embryo count as 264 pg/mL in Japanese Black cattle. On the other hand, in Gir cattle AMH cut‐off value for embryo count was 681 pg/mL (Feres et al., 2024). In Holstein cattle, AMH cut‐off value for corpus luteum was reported as 123.5 pg/mL with 70% sensitivity. However, in this study, AMH cut‐off for total embryo was 227 pg/mL in Holstein cattle with 100% sensitivity. This indicates that serum AMH could be reliably used to select donors.

## 5 CONCLUSION

In summary, this study found significant correlations between AMH levels and superovulation response in donors exhibiting a wide range of AMH levels. In this study, BCS and parity of donors did not affect AMH levels. Furthermore, it was noted that there was no statistically significant difference in the AMH levels of the same animals assessed at two distinct time points. AMH levels are highly repeatable in Holstein cattle. Overall findings indicate that AMH could be used as a reliable marker in determining donors in Holstein cows.

## AUTHOR CONTRIBUTIONS

**Davut Koca**: writing – original draft; visualisation; conceptualisation; investigation; writing – original draft; review & editing. **Ahmet Aktar**: software; writing – original draft; investigation; visualisation; conceptualisation. **Ali Osman Turgut**: software; formal analysis; writing – original draft; review & editing. **Selim Alcay**: investigation; validation; writing – original draft; review & editing; visualisation; funding; conceptualisation; supervision. **Hakan Sagiırkaya**: investigation; visualisation; conceptualisation; supervision; funding acquisition; project administration; validation; methodology; writing – review & editing. All authors approved the final version of the manuscript.

## CONFLICT OF INTEREST STATEMENT

There are no conflicts of interest to declare.

## ETHICS STATEMENT

The study adhered to the approved ethical guidelines of Bursa Uludag University (Approval no. 2021‐13/02). The authors affirm their adherence to the ethical policies of the journal and declare that the study has been conducted in accordance with them.

## Data Availability

The data sets generated for this study are available on request to the corresponding author.
